# Hybridization and differential introgression associated with environmental shifts in a mistletoe species complex

**DOI:** 10.1038/s41598-018-23707-6

**Published:** 2018-04-03

**Authors:** Fernanda Baena-Díaz, Santiago Ramírez-Barahona, Juan Francisco Ornelas

**Affiliations:** 1 Departamento de Biología Evolutiva, Instituto de Ecología, AC, Carretera antigua a Coatepec No. 351, El Haya, Xalapa, Veracruz, 91070 Mexico; 2 Departamento de Botánica, Instituto de Biología, Universidad Nacional Autónoma de México, Tercer Circuito s/n, Ciudad Universitaria, Coyoacán, Ciudad de Mexico, 04510 Mexico

## Abstract

Host specialization after host shifting is traditionally viewed as the pathway to speciation in parasitic plants. However, geographical and environmental changes can also influence parasite speciation, through hybridization processes. Here we investigated the impact of past climatic fluctuations, environment, and host shifts on the genetic structure and patterns of hybridization and gene flow between *Psittacanthus calyculatus* and *P*. *schiedeanus*, a Mesoamerican species complex. Using microsatellites (408 individuals), we document moderate genetic diversity but high genetic differentiation between widespread parental clusters, *calyculatus* in dry pine-oak forests and *schiedeanus* in cloud forests. Bayesian analyses identified a third cluster, with admixture between parental clusters in areas of xeric and tropical dry forests and high levels of migration rates following secondary contact. Coincidently host associations in these areas differ from those in areas of parental species, suggesting that past hybridization played a role in environmental and host shifts. Overall, the observed genetic and geographic patterns suggest that these *Psittacanthus* populations could have entered a distinct evolutionary pathway. The results provide evidence for highlights on the importance of the Pleistocene climate changes, habitat differences, and potential host shifts in the evolutionary history of Neotropical mistletoes.

## Introduction

The speciation process is viewed as a continuum (the ‘speciation continuum’) with many stages that vary in space and time^1–3^, particularly for closely related taxa with varying levels of divergence^4^. The duration of different stages along the speciation continuum depends on the magnitude and timing of gene flow and on the balance among other evolutionary forces including genetic drift, recombination and selection^3,5^, which in turn are likely affected by historical and environmental factors^6^. Understanding population divergence and species boundaries is a complex task because it needs the integration of historical, demographic and ecological data^5,7,8^. For instance, knowledge of historical changes of distributions of populations is essential to understand current geographic patterns of genetic structure and species diversification^9^.

Based on a three-gene mitochondrial DNA phylogeny of seed plants, researchers inferred at least 11 independent origins of parasitism in Angiosperms (1%), eight of which consist entirely of holoparasitic species that lack photosynthetic ability^10^. Modern-day parasites have disproportionately evolved in certain lineages and the endoparasitic habit has arisen by convergence in four clades^10^. In addition, single gene analyses revealed multiple horizontal transfers from host to parasite lineage, making parasitic plants a very interesting model for studying the effects of the parasitic lifestyle on plant diversification^10^. Mistletoe parasitism has been postulated as a major driver of speciation because the parasite’s success depends on multiple, sometimes specialized biotic interactions (hosts, pollinators and seed dispersers) that could indirectly promote the strength and evolution of traits reinforcing the long-term relationship between species enough to isolate parasite populations into distinct races^11–19^.

The parasitic Loranthaceae (Santalales) are mostly aerial mistletoes very common in temperate and tropical plant communities, and typically use a broad range of host tree species^15,20–22^. The life cycle of most loranth mistletoes starts with frugivorous birds dispersing the seeds from tree to tree. Once the seeds are regurgitated or defecated by the bird, these adhere to the host tree with the natural ‘glue’ viscin, and then penetrate the woody host tissue with a structure named haustorium^13,15^. Upon reaching sexual maturity, the pollen is typically moved from flower to flower by a wide range of pollinators, including several species of insects, birds and bats^16,23–27^. Given that most avian seed dispersers and pollinators of loranth mistletoes are not sufficiently specialized^21^, it is unlikely that gene dispersal vectors reproductively isolate mistletoes into populations growing on different hosts within a community (for alternative scenarios see^16,28,29^). Instead, the genetic structuring of mistletoe populations is more likely to be influenced by host-parasite interactions^12,30–32^.

Mistletoes are expected to establish and survive on higher-quality hosts (‘host quality’ hypothesis^33^) and, therefore, variation in host quality would account for non-random occurrence patterns of parasitic plants. Beyond these mechanisms determining the distribution at local and large geographical scales, the diversification of mistletoe species has been explained through different mechanisms linked to host-parasite interactions^12^. Accordingly, the most accepted explanation of mistletoe diversification is that of ‘host-race formation’^12,34–36^, where genetic differentiation, and eventually host-race formation, is acquired through isolation-by-distance or by ecological adaptation following the ‘invasion’ of a different host species (‘host-switching’ hypothesis^12^). In addition, the geographic structuring of genetic variation in some mistletoe species has been explained as the result of past climate changes^31,32,37,38^, landscape fragmentation^39^, emergence of biogeographic barriers^30–32,38^, and by the parasites’ own climatic niche preferences^40,41^.

Although hybridization and introgression have been acknowledged as potentially important mechanisms for adaptive evolution^42–44^, their effects on the genetic structuring of mistletoe populations remain unexplored. Under changing climatic conditions, hybridization and introgression could have played an important role in diversification by enhancing species’ responses to environmental changes^45^. More specifically, the strong impact of Pleistocene climate cycles on distributions of populations of North American plant communities^46–50^, provided the opportunity for populations of recently diverged species to come into secondary contact, leading to hybridization, introgression, and the presence of heterogeneous admixed populations^11,51–54^. In parasitic species, the presence of hybrid intermediates can affect patterns of host specificity by facilitating host shifts, enhancing virulence or increasing the transmission rates of among different hosts (‘hybrid bridge’ hypothesis^55,56^). Thus, the evaluation of the temporal and geographical patterns of hybridization in mistletoe species may shed light onto complex evolutionary scenarios of species divergence and their association with host preferences and geographic isolation of populations.

*Psittacanthus* (Loranthaceae, *c*. 119 species) are characteristic stem parasites (hemiparasites) throughout the Neotropics^21^. Most species are host generalists and ecologically very important because they provide food resources (e.g., fruits and nectar) to many animals^13^ and indirectly influence community structure in low productivity systems^33,57^. The recently diverged species complex of *Psittacanthus calyculatus* and *P*. *schiedeanus* (*c*. 2.5–1.8 Ma^32,38^) can be found infecting different host tree species under distinct environmental conditions along their wide geographical distributions^29,41,58–60^. Kuijt^21^ reported that these species are distributed, more or less sympatrically, from Mexico to Panama, but recent molecular data suggest that these are allopatric and restricted to Mexico^41^. In addition, *P*. *calyculatus* mainly parasitizes *Quercus* (Fagaceae) trees in the central highlands of Mexico, whereas *P*. *schiedeanus* parasitizes cloud forest trees, mainly *Liquidambar styraciflua* (Altingiaceae) in eastern Mexico^32,38,41^. Patterns of genetic structure in chloroplast DNA (cpDNA) sequences and nuclear microsatellites showed two main lineages within the species complex^32,38,41^, with further genetic structuring of populations and evidence of genetic admixture in particular regions^41^. However, the presence of highly admixed individuals within particular populations has not been explored or discussed further^41^. Despite clear geographic and ecological differences between the ‘*calyculatus*’ and ‘*schiedeanus*’ genetic clusters, molecular evidence suggests population admixture within the Tehuacán-Cuicatlán Valley and the Central Valleys of Oaxaca, and the Central Depression of Chiapas^32,38,41^. Overall, these regions have a drier climate and more xeric vegetation types than those occurring elsewhere at higher elevations. Interestingly, populations within these two regions tend to infect a different set of host species^16,21,41^. Furthermore, past and present distribution models predict geographic overlap between the two species in the Central Valleys of Oaxaca during the last glacial cycle (ca. 100–200 kya^32,38^). Therefore, we would hypothesize that past migrations of the two species into the more xeric lowlands led to a secondary contact zone, possibly associated with shifts in host-preferences.

In this study we re-analysed previously reported microsatellite data^41^ to estimate levels of hybridization among groups of populations and rates of migration and directionality over contemporary and historical timescales. We then inferred the most likely scenario and timing of secondary contact and introgression using historical demography and Approximate Bayesian Computation (ABC) methods. Specifically, we addressed the following questions: (1) Are levels of hybridization and introgression geographically concordant with differences in the climatic and host preferences of populations? (2) Are the predicted temporal changes in migration and range shifts associated with the presence of varying levels of hybridization and introgression? We discuss the main causes that could account for the complex phylogeographic patterns observed and the ecological context that could provide valuable clues for the understanding of the evolutionary course of introgressive hybridization in a parasitic plant species’ complex.

## Results

### Genetic differentiation and population structure

Descriptive statistics for the two groups defined by previous species assignments (CALY and SCHI) were remarkably similar to groups defined by habitat, admixture level or geography (Table S2), suggesting shared patterns of demographic history between the CALY and SCHI groups. Allelic richness was generally high for all groups, yet the observed heterozygosity (*H*_*O*_) was lower than expected and inbreeding coefficient values ranged from 0.038 to 0.64 (Table S2). The existence of two major clusters was supported by AMOVA, in which 5.8% of the total genetic variation was explained by significant differentiation between the CALY and SCHI genetic groups (Table S3). When AMOVA was used to explore for geographic structure between groups separated by habitat (three groups), admixture (three groups) or geography (four groups), group differences contributed significantly to 5.9%, 6.6% and 6.38% of the total variance, respectively (Table S3).

Genetic differentiation (*F*_ST-NA_) and allele frequencies (Jost’s *D*) showed the same pattern of differentiation among groups (Table 1). The highest values of genetic differentiation were observed between populations from the TMVB and SMOr, corresponding to group-comparisons by species and admixture level (CALY vs. SCHI) and habitat (XTF vs. CF) (Table 1). Interestingly, the CALY and SCHI groups are clearly differentiated from each other and the HYBR group was more similar to CALY (Table 1).

**Table 1:** Genetic differentiation (*F*_ST_) corrected by the presence of null alleles and absolute allele frequencies differences (Jost’s *D*) between groups with 95% CI.

	*F*_ST_-ENA	Jost’s *D*
**Species**		
CALY vs. SCHI	0.107 (0.055–0.183)	0.61 (0.57–0.65)
**Habitat type**		
XTF vs. CF	0.133 (0.074–0.214)	0.64 (0.60–0.68)
XTF vs. TDF	0.076 (0.044–0.129)	0.56 (0.51–0.62)
CF vs. TDF	0.058 (0.030–0.111)	0.27 (0.20–0.35)
**Admixture level**		
CALY vs. HYBR	0.082 (0.044–0.153)	0.53 (0.47–0.58)
CALY vs. SCHI	0.152 (0.068–0.264)	0.60 (0.56–0.65)
SCHI vs. HYBR	0.101 (0.066–0.148)	0.65 (0.57–0.70)
**Geography**		
TMVB vs. OAX	0.084 (0.045–0.155)	0.48 (0.41–0.53)
TMVB vs. SMOr	0.157 (0.077–0.260)	0.61 (0.56–0.65)
TMVB vs. CHIS	0.113 (0.067–0.174)	0.67 (0.58–0.76)
OAX vs. SMOr	0.117 (0.082–0.165)	0.68 (0.60–0.75)
OAX vs. CHIS	0.074 (0.046–0.110)	0.54 (0.45–0.64)
SMOr vs. CHIS	0.099 (0.049–0.168)	0.50 (0.39–0.61)

TMVB = *calyculatus* group, SMOr = *schiedeanus* group, OAX = Oaxaca region, CHIS = Chiapas region. CF = cloud forest, XTF = temperate forest and xeric groups, TDF = Tropical dry forest. HYBR = admixed populations from Oaxaca and Chiapas regions. Estimates were done over 9 loci.

### Admixture analysis and hybrid identification

**Figure 1:**
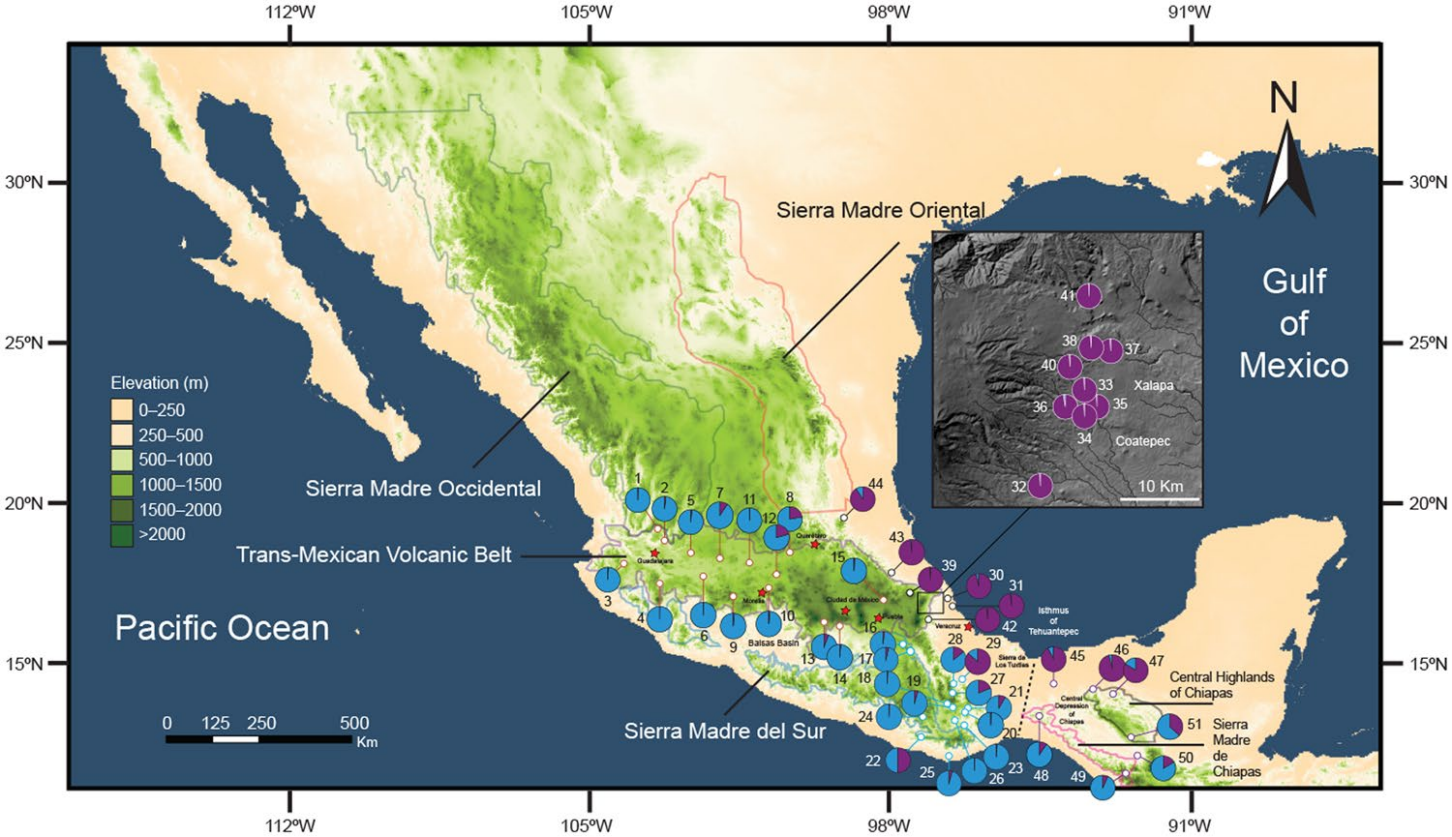
Relief map showing collection sites and assignment probabilities of individuals to populations of the *Psittacanthus calyculatus*/*P*. *schiedeanus* complex in Mexico. In the inset are the collection sites located in central Veracruz. Numbers refer to collection sites according to Table S1. Average assigning probabilities of individuals to putative populations at *K* = 2 according to STRUCTURE analysis (see Results). Pie colour coding is the same as in Fig. 2. The studied populations are located in the map with specific colours corresponding to the genetic groups: CALY = *P*. *calyculatus* (blue), SCHI = *P*. *schiedeanus* (purple). Stars represent main cities along the Trans-Mexican Volcanic Belt. The Mexican mountain systems are highlighted by contour lines corresponding to Sierra Madre Occidental (dark green), Sierra Madre Oriental (orange), Trans-Mexican Volcanic Belt (violet), Sierra Madre del Sur (light blue), Sierra Madre de Chiapas (pink), and Central Highlands of Chiapas (black). This map was generated using the ‘raster’ package in R (https://CRAN.R-project.org/package=raster) and the Global 30 arc-second elevation (GTOPO30) model at a 30-arc seconds spatial resolution (c. 1 km) developed with data through a collaborative effort led by the U.S. Geological Survey’s (USGS) Center for Earth Resources Observation and Science (EROS) Center (https://lta.cr.usgs.gov/GTOPO30). The map in the inset is based on digital elevation model (DEM) available from the Instituto Nacional de Estadística y Geografía (INEGI; http://www.inegi.org.mx/). The figure was drawn using Adobe Illustrator CS6 v16.0.0 (Adobe Systems, Inc.).

**Figure 2:**
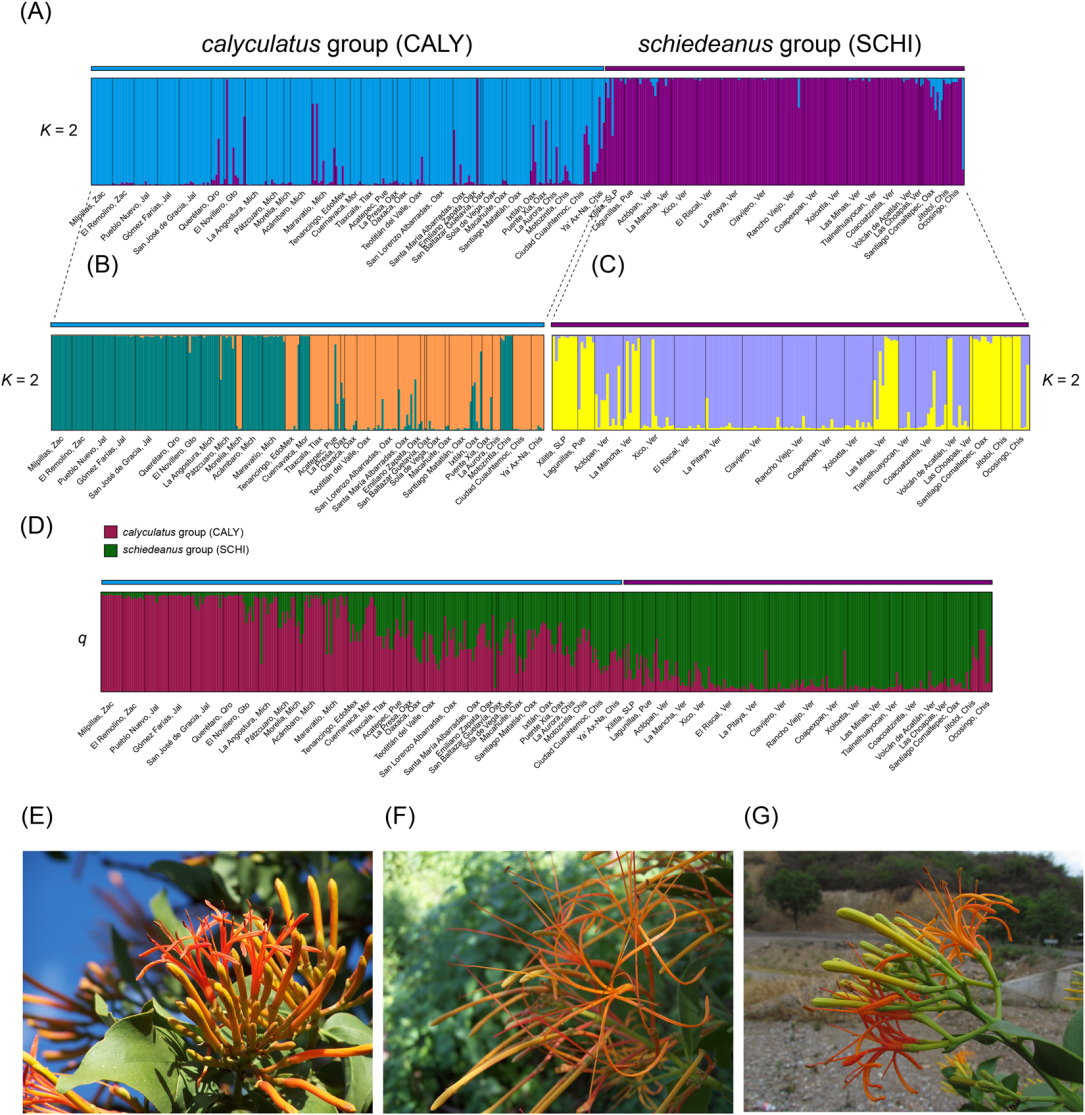
Assignment probabilities (*q*) of 408 *P*. *calyculatus/schiedeanus* individuals at *K* = 2 using STRUCTURE (**A**), and assignment probabilities of individuals to putative sub-structure at *K* = 2 within each first-level clustering, with individuals of the *P*. *schiedeanus* (**B**) and *P*. *calyculatus* (**C**) excluded. (**D**) Assignment probabilities of individuals to pure species (*P*. *calyculatus*, *P*. *schiedeanus*) using the POPFLAG prior information and correlated allele frequencies. Each individual is represented by a vertical line that is partitioned into coloured sections, with the length of each section proportional to the estimated membership coefficient. Photos show flowers of (**E**) *Psittacanthus calyculatus* (by Eduardo Ruiz Sanchez) from Jalisco, (**F**) *Psittacanthus schiedeanus* (by Juan Francisco Ornelas) from Veracruz, and (**G**) *Psittacanthus* putative hybrid (by Eduardo Ruiz Sanchez) from the contact zone in Oaxaca. Note their morphological similarities, with *P*. *schiedeanus* larger leaves and fruits, and flowers of typically much longer and more slender than flowers of *P*. *calyculatus*^21^. Flower size is variable in *P*. *schiedeanus* and sometimes appears to be intermediate to *P*. *calyculatus*.

As expected, two genetic groups were recovered by STRUCTURE (best-supported *K* = 2 determined by the Evanno’s method^61^; Figs 1 and 2A), partially corresponding to previously recognized cpDNA lineages, *P*. *calyculatus* and *P*. *schiedeanus*^32,38,41^. Further sub-structuring was observed within each of the two clusters, in the *P*. *calyculatus* sub-structure corresponded to populations from the western-central portion of the Trans-Mexican Volcanic Belt (TMVB) and populations from central Mexico (Tlaxcala, Puebla) and Oaxaca (Fig. 2B), whereas in the *P*. *schiedeanus* sub-structure corresponded to populations from the Sierra Madre Oriental (SMOr, central Veracruz) and populations from the northernmost distribution (Puebla and San Luis Potosí), Oaxaca, and Chiapas (Fig. 2C). At *K* = 2, several individuals showed signs of admixture and were identified as individuals with an apparent hybrid ancestry between the two main clusters (assignment uncertainty).

**Figure 3:**
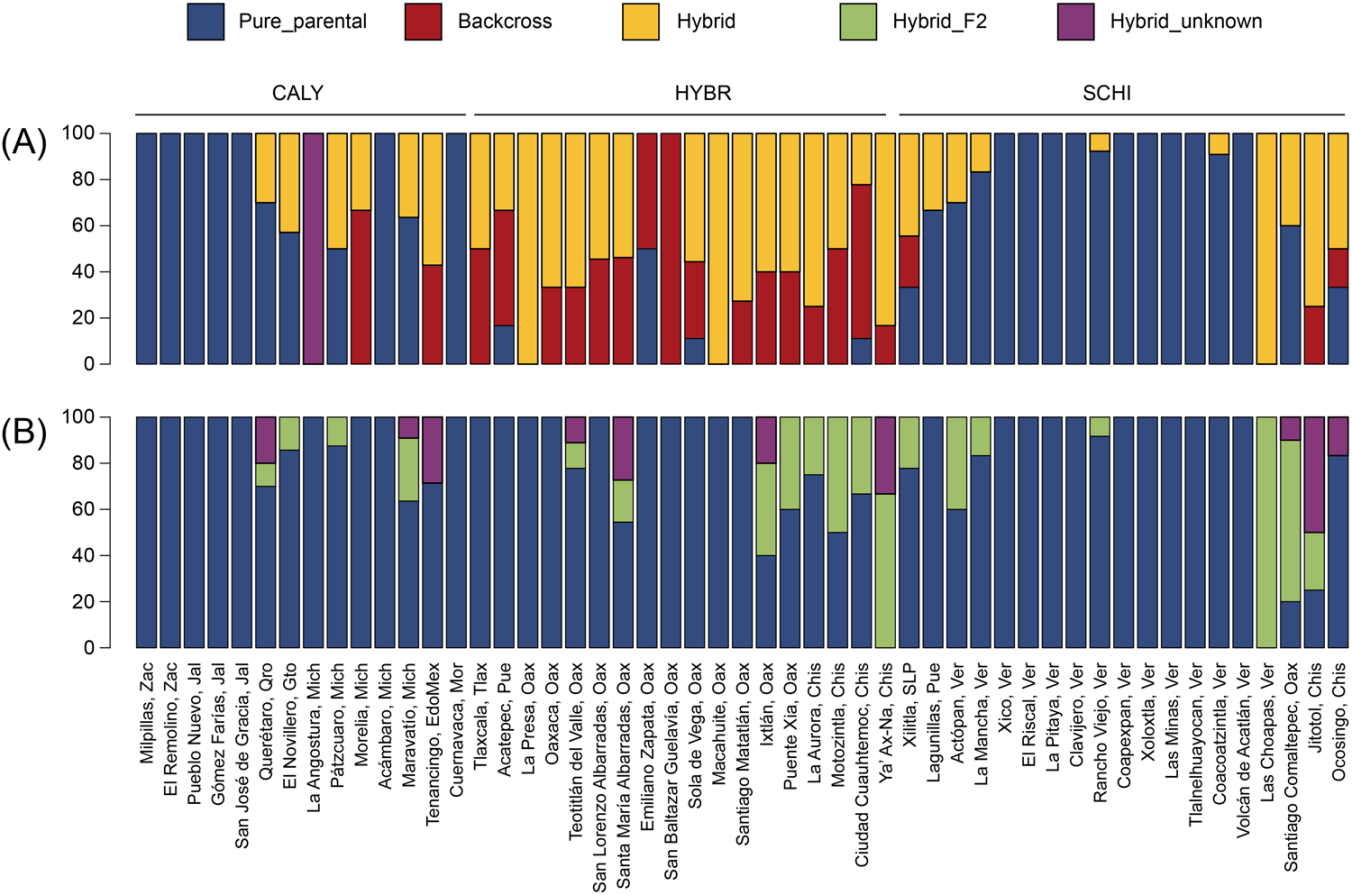
Percentage of hybrids in each population estimated with (**A**) STRUCTURE and (**B**) NEWHYBRIDS. CALY = *calyculatus* group, SCHI = *schiedeanus* group, OAX = Oaxaca region, CHIS = Chiapas region.

Admixture analysis using STRUCTURE revealed high levels of interspecific admixture in the Central Valleys of Oaxaca and the Central Depression of Chiapas (Fig. 2D). In general, we found a higher proportion of individuals with some degree of hybridization in these two regions, compared to populations from the TMVB (CALY) and the Sierra Madre Oriental (SCHI) (Fig. S1 in Supporting information). When comparing group assignments to the simulated data, we found that several populations varied in the percentage of individuals belonging to each hybrid category (Parental, Hybrid and Backcrosses; Fig. 3). NEWHYBRIDS identified fewer hybrid individuals and these were classified as F2 hybrids or unknown. No F1 or first generation backcrosses were found and some individuals were classified as hybrids of unknown hybrid origin (latter generation hybrids or generation backcrosses; Fig. 3).

### Contemporary and historical migration rates

BAYESASS runs yielded low levels of contemporary gene flow between the two main genetic groups (<10%). The highest migration rates were inferred from the CALY population to the SCHI and HYBR populations and from the SCHI population to the CALY and HYBR populations (Table 2). Migration rates from some populations were not significantly different from zero and migration between groups was very low (<2%).

**Table 2:** Contemporary and historical migration rates estimated with BAYESASS and MIGRATE between the two species (CAL = *P*. *calyculatus*, SCHI = *P*. *schiedeanus*) and the hybrid (HYBR) region (OAX = Oaxaca region and CHIS = Chiapas region). CI 95% intervals are given for contemporary migration (*m*). Mean and 25% and 75% posterior distribution percentiles of are given for historical migration (M).

Recipient population	Source population		
	CALY	HYBR	SCHI
**BAYESASS**			
Recent migration rates (*m*)			
CALY	0.9910 (0.0051)	0.0034 (0.0033)	0.0026 (0.0025)
HYBR	0.0048 (0.0038)	0.9866 (0.0072)	0.0037 (0.0038)
SCHI	0.0037 (0.0034)	0.0101 (0.0065)	0.9937 (0.0045)
**MIGRATE**			
Historical migration rates (M)			
CALY	—	0.12 (0–0.63)	0.36 (0.15–0.87)
HYBR	0.23 (0.03–0.78)	—	0.36 (0.12–0.90)
SCHI	0.11 (0–0.6)	0.20 (0–0.36)	—
Historical migration rates (*m*)			
CALY	—	0.00006 (0–0.0003)	0.0002 (0.00007–0.0004)
HYBR	0.0001 (0.00001–0.0003)	—	0.00018 (0.00006-0.0004)
SCHI	0.000055 (0-0.0003)	0.0001 (0–0.00018)	—
Migrants per generation (θ × M)			
CALY	—	1.40 (0–8.6)	2.06 (0.36–3.05)
HYBR	1.79 (0.3–6.1)	—	2.03 (0.29–3.15)
SCHI	0.83 (0–4.7)	2.37 (0–4.9)	—

Theta values: CALY (θ1) = 7.56; HYBRID (θ2) = 11.49; SCHI (θ3) = 5.56.

Estimates of historical migration rates (*M*) calculated using MIGRATE revealed generally low migration rates among populations, except for the slightly higher migration rates from the SCHI population to the CALY and HYBR populations and from the CALY population to the HYBR population. Estimates of scaled migration rates (*m*) ranged from 0.00018 to 0.00005 and showed the same pattern among populations. The number of migrants per generation (*N*_e_*m*) ranged from 0.83 to 2.37 (Table 2). Despite the low migration rates, the Mantel test showed that the historical and contemporary migration matrices were not significantly correlated (*r* = 0.0196, *P* = 0.5), implying that the rate and intensity of migration have changed over time between groups, with increased gene flow from past to present.

**Figure 4:**
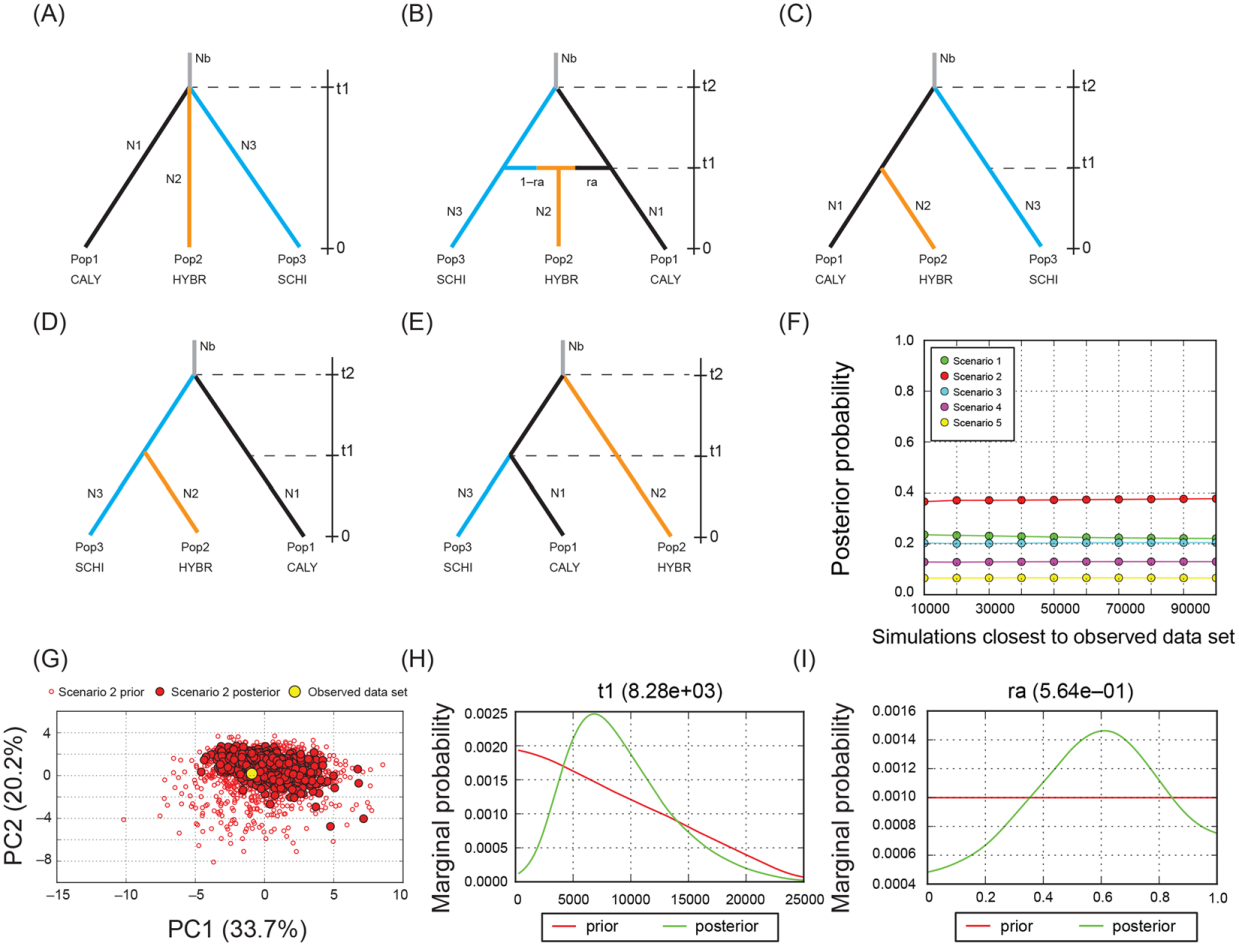
Competing demographic scenarios of *Psittacanthus calyculatus* divergence. Five evolutionary scenarios were built and tested using DIYABC: (**A**) *simple split model* (scenario 1), in which CALY (Pop1), HYBR (Pop2) and SCHI (Pop3) diverged simultaneously at t1; (**B**) *isolation with admixture model* (scenario 2), in which Pop2 (HYBR) was generated by admixture between Pops 1 (CALY) and 3 (SCHI) at t1, then CALY merged with SCHI at t2; (**C**) *hierarchical split model 1* (scenario 3), in which HYBR merged with CALY at t1, then both populations merged with SCHI at t2; (**D**) *hierarchical split model 2* (scenario 4), in which HYBR merged with SCHI at t1, then both populations merged with CALY at t2; (**E**) *hierarchical split model 3* (scenario 5), in which CALY merged with SCHI at t1, then both populations merged with HYBR at t2. The posterior probability of scenarios was assessed using a weighted logistic regression on the 1% of simulated datasets closest to the observed data and, for the best-supported scenario (scenario 2); (**F**) Results of a logistical model comparing the posterior probability of each scenario with the number of simulations used to calculate it; (**G**) A model checking procedure was applied using a PCA on test statistic vectors to visualize the fit between the simulated and observed datasets. Note the large cloud of data from the prior and observed datasets centred on a small cluster from the posterior predictive distribution, suggesting that the best-supported scenario explained the observed data well. Prior and posterior probabilities of parameters t1 (**H**) and ra (=admixture rate) are provided (**I**).

**Table 3:** Posterior probability and 95% confidence intervals (CI) using a logistic regression in the Approximate Bayesian Computation (ABC) analysis for five scenarios of *Psittacanthus calyculatus*/*schiedeanus* divergence and hybridization. Type I and Type II error rates were estimated for scenario 2.

Scenario	Posterior probability	95% CI	Type I error rate	Type II error rate
Simple split model (scenario 1)	0.2212	0.2170–0.2255		
Isolation with admixture model (scenario 2)	**0**.**3778**	**0**.**3729–0**.**3828**	**0**.**28**	**0**.**058**
Hierarchical split model (scenario 3)	0.2000	0.2008–0.2029		
Hierarchical split model (scenario 4)	0.1300	0.1266–0.1335		
Hierarchical split model (scenario 5)	0.0659	0.0631–0.0686		

### Inference of divergence and secondary contact scenarios

DIYABC analyses indicated that isolation with admixture is the best-supported scenario (scenario 2; Fig. 4), with a higher posterior probability value and 95% confidence intervals that did not overlap with those obtained for the other scenarios (Table 3). Under this scenario, divergence between CALY and SCHI occurred 192.5 kya (t2) followed by secondary contact, and admixture between CALY and SCHI giving rise to the HYBR populations in the Central Valleys of Oaxaca and Central. Depression of Chiapas. Posterior mean parameter estimates suggest that secondary contact occurred 9,010 generations ago (t1). Considering an 11-yr generation time for *Psittacanthus*^32^, this translates into 99.1 kya (Table S4 in Supporting information), consistent with a period in which the ranges of CALY and SCHI are predicted to overlap. Finally, the most probable admixture mean rate (*ra* = 0.547) was higher between the CALY and HYBR populations than admixture between the SCHI and HYBR populations (1-*ra* = 0.453; Table S4 in Supporting information).

## Discussion

### Patterns of genetic structure in the *Psittacanthus* species complex

We investigated patterns of genetic structure and levels of hybridization among groups of populations and estimated rates and directionality of migration over contemporary and historical timescales between *P*. *calyculatus* and *P*. *schiedeanus* using nuclear microsatellites. Our study revealed that the high levels of admixture are best explained by past introgression as a result of gene flow during secondary contact.

The distribution of individuals with signs of admixture was not restricted to the area of sympatry in the Central Valleys of Oaxaca, but also occurred in the Central Depression of Chiapas, which is separated by warmer and drier conditions along the Isthmus of Tehuantepec from most other populations of the species^41^. These two regions presented a higher proportion of intermixed individuals, likely F2 or later generation hybrids. Although genetic differentiation between *P*. *calyculatus* and *P*. *schiedeanus* is moderate (*F*_ST-NA_ = 0.107), the genetic differentiation between groups increases (*F*_ST-NA_ = 0.152) when data for populations of the HYBR group are excluded, indicating that this group represents a genetic bridge between the two parental species. Overall, the admixture results confirm our prediction that the more arid Central Valleys of Oaxaca and Central Depression of Chiapas represent hybrid zones. However, the genetic distinction between parental species can be maintained by host association (environmental context) and by the geographic characteristics of their ranges, even in the presence of gene flow.

Interestingly, the very low genetic differentiation and high admixture levels between populations in the Central Valleys of Oaxaca and the Central Depression of Chiapas suggest that the Isthmus of Tehuantepec may not be strong barrier to gene flow for these mistletoes, as observed for other bird-dispersed plant species^62,63^, but not in other species with different dispersal mechanisms^48,64–66^. In addition, the Chiapas region (and adjacent Guatemala) is also home to other species of *Psittacanthus* closely related with *P*. *calyculatus* and *P*. *schiedeanus*^41^: *P*. *breedlovei* and *P*. *angustifolius*^21^. Thus, it is possible that hybridization with one of these other species may be the reason for the apparent high admixture and the low genetic differentiation between mistletoes in these two regions. However, these two species can be easily recognizable based on leaf morphology and host preferences^21^. In addition we observed, albeit using few samples, that *P*. *angustifolius* and *P*. *breedlovei* appeared to be genetically indistinguishable from their widespread sister species^41^. Therefore, further exploration of the genetic patterns including more samples from the other *Psittacanthus* species distributed in Chiapas is needed to understand their evolutionary history within this region.

### Species boundaries and differential admixture

The observed genetic structure and migration patterns seem to be linked to the demographic history of the species complex. For instance, migration rates clearly increased from past to present between geographical regions. Migration rates showed that historical and contemporary gene flow was directional from the SCHI and CALY populations to the HYBR population, whereas contemporary gene flow occurred also from the CALY population to the SCHI population (Table 2). The low differentiation between the HYBR populations in Oaxaca and the CALY group might be due to asymmetrical gene flow, resulting in higher levels of introgression from *P*. *schiedeanus* into *P*. *calyculatus* (Fig. 2d). In theory, minimal differences in the timing of flowering or fruiting phenologies may promote pre-mating isolation and prevent hybridization between *P*. *schiedeanus* and *P*. *calyculatus*. However, the flowering and fruiting periods of the two species in the arid Central Valleys of Oaxaca greatly overlap^23,24,58,60^. Thus, further mechanisms for such considerable degree of gene flow have to be considered.

Díaz Infante and collaborators^16^ investigated the reproductive biology and phylogenetic relationships between a population of *P*. *calyculatus* from the Trans-Mexican Volcanic Belt and two sympatric populations of *P*. *calyculatus* and *P*. *auriculatus* from the xeric Central Valleys of Oaxaca. Flowers of the two species in sympatry were reciprocally pollinated to assess post-mating components of reproductive isolation (*RI*), with fewer heterospecific matings observed than expected by chance in *P*. *calyculatus* compared to *P*. *auriculatus*. When considering other factors of ecological isolation that affect co-occurrence, the *RI* values indicated that isolation by hummingbird pollinators was less effective than isolation by host tree species and seed dispersers, suggesting that host usage is the most important ecological isolation factor between the two species and that the host tree species’ barrier is currently contributing the most to maintaining these species in sympatry^16^.

For the origin of the hybrid forms located in the Central Valleys of Oaxaca and the Central Depression of Chiapas regions, a late Pleistocene scenario and assumed hybridization processes seem to be a plausible alternative for some reasons. The most likely DIYABC scenario supported a hybrid origin of the populations occurring c. 99 k years ago, corresponding approximately to the early last glacial period (Wisconsin glaciation; 120–110 ka). This is consistent with the estimated initial diversification of the species complex occurring 200 kya^32^, followed by dramatic changes in the distribution of species during the last glacial period^32,38^. Under this scenario, populations of the *Psittacanthus calyculatus* and *P*. *schiedeanus* may have expanded into the currently xeric lowlands of Oaxaca during glacial periods, with secondary contact and formation of hybrid zones among otherwise genetically differentiated populations (sympatric stage). During the Holocene, populations of the two parental species contracted back into temperate and cloud forests at higher elevation where little genetic structure is observed (allopatric stage). Alternatively, ancestral hybrid populations of *P*. *calyculatus* and *P*. *schiedeanus* remained *in situ* (in the arid Central lowlands of Oaxaca and Chiapas) and differentiation of these populations occurred under more xeric conditions and specializing on different host species (host shifting hypothesis).

### Genetic status of the Oaxaca populations

Individuals with signatures of admixture were most frequent in populations located in the central arid regions of Oaxaca and Chiapas (HYBR group). The observed historical and contemporary patterns of gene flow shed some light on how historical gene flow between these closely related species occurred and what geographical barriers have maintained their genetic integrity. The non-significant correlation between historical and contemporary migration rates implies that migration rates between groups have changed over the evolutionary history of the species complex. Although rates of historical and contemporary migration were low among populations, we found evidence of past migration from the CALY and SCHI populations into the HYBR populations, supporting the demographic model for the hybrid origin of these populations. Results of the DIYABC analysis supported one of the allopatric speciation scenarios and indicated that divergence between groups of populations occurred at least 100 kya, consistent with previous evidence using nuclear and chloroplast DNA sequences^32,38^. Although we tested several scenarios of speciation with gene flow, future work incorporating genomic data might give more accurate estimates of the dynamics of gene flow and test alternative and more complex scenarios of allopatric speciation followed by hybridization during secondary contact^67^.

Previous phylogeographic studies using cpDNA sequences, accompanied by ecological niche modelling, provided evidence that individuals of *P*. *schiedeanus* and *P*. *calyculatus* from the Central Valleys of Oaxaca formed a genetic cluster different from those corresponding to the distribution of each of the two species, and recognized this region as a potential area of secondary contact^32,38^. Our study revealed that the arid Central regions of Oaxaca, as well as the Central Depression of Chiapas, represents a secondary contact hybrid zone between *P*. *calyculatus* and *P*. *schiedeanus*. An interesting feature of the overlapping distribution area in Oaxaca for these mistletoes is that the more xeric climatic conditions contrast with the adjacent pine-oak forests (*P*. *calyculatus*) and cloud forests (*P*. *schiedeanus*) that parental species inhabit^32,38,41^. In addition, the main host species used by HYBR individuals in the secondary contact zone are also different, with a higher prevalence (57%) on *Celtis caudata* (Ulmaceae) at one locality^16^ or strongly associated with Anacardiaceae hosts based on herbarium records^21,41^. Therefore, we propose that expansion of *P*. *calyculatus* and *P*. *schiedeanus* towards lower elevations during the last glaciation^32,38^, promoted different events of hybridization in secondary contact zones, creating a hybrid swarm, probably with low hybrid frequencies. Once the glaciation period was over, admixed individuals prevailed within the same area because these had the capacity to confront new environmental conditions with different host species, with drier climatic conditions and more xeric vegetation, whereas non-admixed individuals contracted into the highlands to their present distribution along the TMVB and SMOr associated with different sets of host species. This hypothesis does not exclude the possibility of current gene flow (though restricted) to areas where cloud forests are very close to the tropical dry forests and xeric zones, like in the Central Valleys of Oaxaca. Secondary contact and hybridization could have weakened the reproductive barriers and maintained low genetic differentiation between populations from the temperate (TMVB) and cloud forests (SMOr). In light of the weak reproductive barriers between *P*. *calyculatus* and *P*. *auriculatus*, the sister group of the *P*. *calyculatus* and *P*. *schiedeanus* species complex^16,32^, we believe that hybridization very likely occurs among populations within the complex due to close genetic relationships between species. In addition, over the 20 bird species recognized as seed dispersers and more than 20 hummingbird species that pollinate the flowers of these mistletoe species^23,24,58,60,68^, several species are shared^16^ thus increasing the probabilities of current gene flow between species. It is possible that the present hybrid population has resulted from contemporary gene flow between parental species facilitated by bird seed-dispersal, rather than the result of historical gene flow by secondary contact after the differentiation of the two parental *Psittacanthus* species in late Pleistocene. Our analyses showed that contemporary gene flow is present between the parental and the hybrid genetic clusters. However, we believe that if hybrid populations result only from recent gene flow, we would also have observed a significant proportion of pure parental individuals within the hybrid zone. That is, birds would transport (in independent events) pure parentals into one region and only after their establishment would these begin to hybridize. Nonetheless, first generation hybrids were almost absent in the analyses and the timing of admixture by the DIYABC analysis was congruent with the idea of secondary contact after the differentiation of the two parental *Psittacanthus* species in late Pleistocene.

In parasitic plants the colonization of a new environment and further differentiation could be associated to adaptations to biotic factors such as new host associations, pollinators and dispersers or through adaptations to abiotic factors (like the parasite’s own niche) or both^41,69^. In mistletoes, controversial evidence exists about the role of host race formation as the main diversifying force. Here gene flow can be interrupted or diminished if mating, dispersal and establishment occur only among mistletoes adapted to specific hosts^36^. Particularly, cross-dispersal experiments have provided some evidence for host-race formation in *P*. *schiedeanus* and *P*. *calyculatus* growing on distantly related host species in sympatry, in which seedling development was greatest when seeds were placed on their source host species^60,70^. The hypothesis of host-race formation is supported by the observed low heterozygosity values that might be produced by subpopulation structure, i.e. mistletoes that aggregate in one or adjacent hosts by geographic or behavioural barriers of gene dispersal vectors to gene flow followed by genetic drift in the subpopulations (Wahlund effect^14^). The significant population differentiation and genetic structure in the *P*. *calyculatus* and *P*. *schiedeanus* species complex has been attributed to climatic variables, rather than to host association^41^. Evidence in plant, fungi and animal species has shown the potential of hybridization to the adaptation to different environmental conditions^44,71–73^. In our study, we found that the area with higher values of admixture corresponds to individuals from locations with drier climatic conditions, more xeric vegetation types and different associated host species, suggesting that colonization of a ‘new’ habitat is linked to hybridization and further genetic differentiation.

With the evidence in hand, many questions arise about the role of hybridization, reinforcement and introgression for the evolution of this system, including the colonization of new environments, host shifts, host race formation, and a possible case of hybrid speciation. If the reproductive barriers are weak and there is no reinforcement (selection against hybrids), differentiation between species will be eroded and the effect of host race formation would be diluted^74^. On the other hand, strong selection to different environments could maintain genetic differences between populations, despite the presence of gene flow^1,3^. Further studies combining genomic scans and experimental data of host-specific relationships through cross-inoculation experiments, or reciprocal transplants to different environments comparing parental and hybrid individuals would shed light on the degree of reproductive isolation and the role of introgressive hybridization in the evolution of host specialization and environmental shifts in *Psittacanthus* mistletoes in particular, and other aerial parasites in general. The processes of reinforcement, introgression and transgressive segregation along with evolutionary forces like genetic drift and selection could influence the chances that the hybrid population adapts to new host species. For example, the gain of alleles in the hybrids through introgression and the presence of transgressive traits (those exceeding the values from parental species) could benefit hybrid individuals to adapt to the new environment by creating a phenotype able to colonize a new host or tolerate different temperatures. Also, if hybrid mistletoes tend to aggregate in different hosts, the role of genetic drift will could be important to reproductively isolate hybrids^3^. We do not yet know the specific forces leading the hybrid populations into a new host as the initial stage towards speciation. It maybe directly related to the hybrid origin, by gaining some genetic advantage permitting the invasion of a new host. Or perhaps the adaptation into a new host is related to the *in situ* survival of the hybrids in the region, which was subjected to floristic turnover during the last-glacial/Holocene transition. In this case, the increasing abundance of arid-adapted hosts (such as Anacardiaceae) would lead to the specialization of these mistletoes and the host shifting leading to host race formation^12^. Results of this study suggest that hybridization could have been important for the colonization to a new environment under past climatic changes and could help to understand the range of outcomes of climate alterations and recently human-altered environmental changes shaping current patters of diversity.

## Methods

### Study sites and molecular data

Our analyses are based on the molecular data provided by Ramírez-Barahona and collaborators^41^. These data included information of 10 microsatellite loci for 415 individuals in 54 populations throughout the species’ distribution ranges in Mexico^41^, an area spreading about 14° in longitude and 10° in latitude, that includes three biogeographical regions. However, due to the few individuals genotyped for some populations we pooled populations in close proximity, ending up with 51 populations for this study (Table S1). The sampling included individuals from allopatric populations from the TMBV and eastern slopes of the SMOr, individuals from sympatric populations in the Central Valleys of Oaxaca and the Central Depression of Chiapas, and individuals of the range-restricted *P*. *breedlovei* and *P*. *angustifolius* in Chiapas (Fig. 1).

All the individuals included in the dataset were successfully genotyped for at least five of the 10 microsatellites, with 70% of the individuals successfully genotyped for seven or more loci. Microsatellite data are available in the Supporting Information of Ramírez-Barahona and collaborators (nph14471-sup-0003-NotesS2.csv)^41^. To confirm the presence of genetic clusters in the sample, we carried out a two-step Bayesian Markov chain Monte Carlo (MCMC) clustering analysis of microsatellite data using STRUCTURE 2.3.4^75^, as previously implemented^41^. The most likely number of populations was determined estimating the Delta*K* (∆*K*) and the log likelihood of *K*, ln P(*K*) = L(*K*) between successive *K* values^61^. According to Evanno’s method^61^, we confirmed the presence of two clusters, CALY and SCHI, and additional genetic sub-structuring was observed within each of these clusters in the second-step analysis (see also^41^ for similar findings). Interestingly, several individuals scattered throughout the STRUCTURE plot show signs of admixture at *K* = 2 (Fig. 2A,C).

### Admixture analysis and hybrid identification

Given the observed patterns of genetic structure at *K* = 2 and the distribution of admixed individuals scattered throughout the STRUCTURE plot, we decided to reanalyse the full dataset particularly because a high number of admixed individuals (mean membership coefficient <0.75) were not included in previous analyses^41^. To explore the occurrence of hybridization (see^76,77^ for more details), we assigned individuals from the allopatric CALY and SCHI clusters as ‘pure parental’ when the membership coefficient (*q*) was *q* > 0.9 for either of the two clusters. Individuals from the sympatric regions (Central Valleys of Oaxaca and the Central Depression of Chiapas) were not considered for the ‘pure parental’ assignment because the suspected high admixture in these regions could bias further assignment. The possibility that *P*. *auriculatus*, geographically restricted to the Oaxaca dry valleys, can be the donor for the hybrid population given its sympatric distribution with the *P*. *calyculatus*/*schiedeanus* complex is unlikely. The specific microsatellites used here^41^ were designed using populations of the species complex of *P*. *schiedeanus* and *P*. *calyculatus* and previous work by our lab group has shown low success in transferring these microsatellite loci into other species due to poor amplification.

We then performed a STRUCTURE analysis using the ‘pure parental’ information, implemented through the POPFLAG prior, to define individuals belonging to the parent populations. In order to uncover admixture between the two species, all individuals were assigned to either CALY or SCHI populations by setting *K* = 2. We set the allele frequencies to be correlated and performed ten replicates with 100 000 MCMC after 50 000 burning period. The outputs of each replicate were combined in CLUMPP for visualization^78^.

We then used the Bayesian model-based program NEWHYBRIDS^79^ to calculate the posterior probability of individuals belonging to one of six categories: (1) pure CALY, (2) pure SCHI, (3) first generation hybrids, (4) second generation hybrids, (5) CALY backcrosses, and (6) SCHI backcrosses. The analysis focuses on first generation hybrids, thus helping to detect on-going hybridization between species. Assignments were done using Jeffrey’s-like prior for allele frequencies and mixing proportions as suggested in the program’s manual. We ran three replicates with 500 000 sweeps and a burn-in period of 20 000, and the prior information of pure parental individuals obtained from previous STRUCTURE included in the analysis.

To define assignment thresholds to each hybrid category used for our results, we performed hybridization simulations in HYBRIDLAB^80^. For this, we used the individuals classified as parental in the STRUCTURE analysis as the starting populations and simulated 50 hybrids for several generations: first, second and third generation hybrids; and first, second and third generation backcrosses to each parental species. Data obtained for each simulation were run in STRUCTURE with the same parameters as above. The results for *K* = 2 were combined with CLUMPP^78^ and the proportion of membership was visualized using R 3.1.3 (R Development Core Team, 2015). From the simulations, we identified three categories of individuals: (1) *pure species*, individuals with an assignment probability of *q* ≥ 0.80 to one cluster; (2) *backcrosses*, individuals with an assignment probability between *q* = 0.6 and *q* = 0.8 to one cluster; and (3) *hybrids* (including the indistinguishable first, second and third generations), individuals with an assignment probability between 0.4 and 0.6 (Fig. S1 in Supporting information).

Finally, the simulated data were also run in NEWHYBRIDS and the program correctly assigned the pure species individuals with a probability >0.9. For the first- and second-generation hybrid individuals, we considered correctly assigned individuals those with probabilities higher than 0.5. Subsequent hybrid generations and backcrosses were not identified in NEWHYBRIDS, thus we considered hybrid individuals of unknown generation those with assignment probabilities between 0.1 and 0.9. Finally, we estimated the proportion of individuals belonging to each category for each population to assess the level of admixture across populations.

### Genetic differentiation and population structure

To analyse genetic differentiation and population structure, we used the classification provided by the initial STRUCTURE runs and grouped populations into two groups, CALY and SCHI. Because of the high levels of admixture observed in the Central Valleys of Oaxaca and the Central Depression of Chiapas, we also grouped samples into three groups as follows: (1) CALY, samples from temperate forests along the TMVB; (2) SCHI, samples from cloud forests in eastern Mexico and three populations in Chiapas; and (3) HYBR, samples from the xeric Central Valleys of Oaxaca and Central Depression of Chiapas. Given that most genetic structure has been explained by environmental factors^41^, we also grouped samples according to habitat type: (1) cloud forests; (2) xeric temperate forests; and (3) tropical dry forests. Finally, populations were also grouped according to geography into four groups: (1) the Chiapas region; (2) the Oaxaca region; (3) Sierra Madre Oriental; and (4) Trans-Mexican Volcanic Belt.

For each of the four groupings (species, geography, admixture, and habitat), we estimated gene diversity and absolute allele frequency differentiation (Jost’s *D*) using the R package “DiveRsity”^81^ and rarefied allelic richness using the R package “hierfstat”^82^. Hardy-Weinberg equilibrium departures and linkage disequilibrium among loci were estimated in GENEPOP 1.2^83^. We used FreeNA^84^ to estimate the frequencies of null alleles with the EM algorithm^85^ and the pairwise genetic differentiation (Weir’s *F*_ST_^86^) among groups of populations using the ENA correction. Note that genetic structure below the level of our sampling (population subdivision) can produce significant departures from Hardy-Weinberg even in the absence of null alleles (i.e. Wahlund effect: reduction of heterozygosity in a population caused by subpopulation structure). For these analyses, locus four was eliminated because it was only present in the SCHI populations.

To explore whether the distribution of the genetic variance in our populations is related to species differences, geography, or to environmental differentiation between groups of populations, we performed an analysis of molecular variance (AMOVA^87^) as implemented in ARLEQUIN 3.01^88^. Four AMOVAs were performed with different groups of populations: (a) two clusters corresponding to each species (CALY and SCHI); three clusters corresponding to (b) habitat type (cloud forest, xeric temperate forest, tropical dry forest) or (c) level of admixture (CALY, SCHI, HYBR); and (d) four clusters corresponding to geography (CHIS, OAX, SMOr, TMVB; Table S1). Significance of each AMOVA was evaluated with 10 000 permutations.

### Contemporary and historical migration rates

We compared migration rates over contemporary and historical timescales^89^ using unlinked microsatellite data with BAYESASS^90^ and MIGRATE^91^ for the three groups identified with the admixture analysis: pure CALY, pure SCHI, and HYBR from the Central Valleys of Oaxaca and the Central Depression of Chiapas. Using Bayesian inference, BAYESASS estimates recent migration rates between populations within the last few generations (*m*), whereas MIGRATE uses the coalescent approach to jointly estimate the relative effective population size θN_e_ (4N_e_µ) and asymmetrical gene flow *M* (m/µ) between pairs of populations over much longer periods of time, approximately thousands of years (ca. 4N_e_ generations in the past^91^).

BAYESASS was initially run with the default delta values (Δ) for allelic frequency (*A*), migration rate (*M*), and inbreeding (*F*). Subsequent runs incorporated different Δ to ensure that the acceptance rate for proposed changes in parameters were between 20–40% for each parameter. Adjusted final delta values used were Δ*A* = 0.2 (41% acceptance rate), Δ*M* = 0.8 (22%) and Δ*F* = 0.2 (48%), respectively. To ensure convergence, we performed five independent runs (50 million iterations, 5 million burn-in, and sampling frequency of 2000) each with a different seed number, comparing the posterior mean parameter estimates for convergence. We also analysed the trace file of each run with TRACER 1.5^92^ to ensure an appropriate mixing of parameters and burn-in number. We give estimates of *m* from one randomly chosen run out of the three final runs as their parameter estimates were similar. We ran MIGRATE incorporating Bayesian inference analyses to estimate historical migration rates (*M*) among groups of populations. We used a Brownian-motion model with a constant mutation rate and *F*_ST_ to estimate θ. Several short runs were performed to search for the appropriate priors. After finding suitable priors, MIGRATE was run three times to confirm convergence. These final runs consisted of one long chain, 100 000 sampled trees, 1000 recorded, with a burn-in of 10000 with three replicates and each run with a different seed number. We set the minimum and maximum boundaries for theta (θ) and migration (*M*) as 0.0 and 30.0, with a delta value of 3. A four-chain heating at temperatures of 1, 1.5, 3 and 10000 was implemented to increase the efficiency of the MCMC^89^. Lastly, we performed a Mantel test with 5000 permutations to test for similarity between contemporary and historical values of *m*. For this analysis, we used the values of *m* directly generated by BAYESASS and estimated *m* from values of *M* (m/µ) generated by MIGRATE by multiplying all *M* values by an estimated mutation rate of 5 × 10^−4^ for microsatellites^93^. The number of migrants per generation was estimated by multiplying the θ value from the source populations to *M*^91^.

### Inference of divergence and secondary contact scenarios

Approximate Bayesian Computation (ABC) analysis was performed using samples in the three clusters defined based on genetic differentiation and genetic sub-structuring: CALY, SCHI, and HYBR. Five possible divergence scenarios leading to these three clusters were included in the ABC analysis to test if the admixed populations have a hybrid origin (see Results). In each scenario, t refers to timescale ranging from 300 to 25000 generations and with a conditional prior t2 > t1, the maximum t included the predicted split between *P*. *schiedeanus* and *P*. *calyculatus* around 2.18 × 10^5^ years ago^32^. The effective size (N) of the corresponding populations (Pops 1, 2, 3 or the ancestral population) during each time period (e.g., 0–t1, t1–t2) was set to a maximum of 30000.

We used DIYABC 2.1^94^ to simulate ten million datasets (2 million per scenario) with the same number of populations, loci and individuals. The most likely scenario was evaluated by comparing posterior probabilities using the logistic regression approach^95,96^. Temporal and demographic parameters were estimated with a logistic regression for the best-supported model with the 1% simulated data closest to the observed data^95,96^. Finally, to assess confidence in the model selection we simulated 500 pseudo-observed datasets (PODs) to estimate type I and type II error rates^95,96^.

### Data availability

The dataset (microsatellites) generated and analysed during the current study are available in the Supporting Information of Ramírez-Barahona *et al*. (nph14471-sup-0003-NotesS2.csv)^41^.

## Electronic supplementary material

Supplementary Information
