# Supplementary material for: Hybridization and differential introgression associated with environmental shifts in a mistletoe species complex
Source: Sci Rep. 2018 Apr 3;8:5591. doi: 10.1038/s41598-018-23707-6 (PMC5882953; doi:10.1038/s41598-018-23707-6)

Supplementary material on

Hybridization and differential introgression associated with environmental shifts in a mistletoe species complex

Fernanda Baena-Díaz[1], Santiago Ramírez-Barahona[1,2] & Juan Francisco Ornelas[1]

[1]Departamento de Biología Evolutiva, Instituto de Ecología, AC, Carretera antigua a Coatepec No. 351, El Haya, Xalapa, Veracruz 91070, Mexico.

[2]Departamento de Botánica, Instituto de Biología, Universidad Nacional Autónoma de México, Tercer Circuito s/n, Ciudad Universitaria, Coyoacán, Ciudad de Mexico, 04510 Mexico.

Correspondence and requests for materials should be addressed to J.F.O. (email: francisco.ornelas@inecol.mx)

Table S1. Geographic location and population codes of the 51 populations from the *Psittacanthus calyculatus* / *schiedeanus* complex used in the study.

Location Code	Species Code	Location	Region	Habitat	*n*	Altitude (masl)	Latitude (N)	Longitude (W)
1	CALY	Mexico, Zacatecas, Milpillas	TMVB	XTF	10	1900	21° 19´ 31´´	103° 36´ 50´´
2	CALY	Mexico, Zacatecas, El Remolino	TMVB	XTF	10	1220	21° 23´ 21´´	103° 07´ 14´´
3	CALY	Mexico, Jalisco, Pueblo Nuevo	TMVB	XTF	11	1545	20° 15´ 30´´	103° 39´ 52´´
4	CALY	Mexico, Jalisco, Gómez Farías	TMVB	XTF	10	1360	19° 52´ 16´´	103° 31´ 47´´
5	CALY	Mexico, Jalisco, San José de Gracia	TMVB	XTF	15	2025	20° 41´ 47´´	102° 33´ 22´´
6	CALY	Mexico, Michoacán, La Angostura	TMVB	XTF	9	1540	20° 12´ 03´´	102° 26´ 35´´
7	CALY	Mexico, Guanajuato, El Novillero	TMVB	XTF	7	1710	20° 27´ 00´´	101° 31´ 58´´
8	CALY	Mexico, Querétaro, Querétaro	TMVB	XTF	10	2700	20° 35´ 00´´	100° 22´ 59´´
9	CALY	Mexico, Michoacán, Pátzcuaro	TMVB	XTF	8	2159	19º 31´ 20´´	101º 36´ 07´´
10	CALY	Mexico, Michoacán, Morelia	TMVB	XTF	3	1921	19° 39´ 00´´	101° 13´ 48´´
11	CALY	Mexico, Guanajuato, Acámbaro	TMVB	XTF	10	1884	20° 02´ 04´´	100° 39´ 45´´
12	CALY	Mexico, Michoacán, Maravatío	TMVB	XTF	11	2231	19° 54´ 06´´	100° 31´ 46´´
13	CALY	Mexico, Estado de México, Tenancingo	TMVB	XTF	7	2031	18° 59´ 28´´	99° 34´ 31´´
14	CALY	Mexico, Morelos, Cuernavaca	TMVB	XTF	5	1380	18° 59´ 06´´	99° 15´ 25´´
15	CALY	Mexico, Tlaxcala, Tlaxcala	TMVB	XTF	16	2200	19° 17´ 00´´	98° 14´ 00´´
16	CALY	Mexico, Puebla, Acatepec-Zapotitlán	OAX	XTF	6	1939	18° 14´ 14´´	97° 33´ 45´´
17	CALY	Mexico, Oaxaca, Cacaotepec, La Presa	OAX	XTF	2	1695	17° 41´ 37´´	97° 36´ 05´´
18	CALY	Mexico, Oaxaca, Oaxaca	OAX	XTF	6	1627	17° 04´ 47´´	96° 39´ 45´´
19	CALY	Mexico, Oaxaca, Teotitlán del Valle	OAX	XTF	9	1680	17° 01´ 29´´	96° 31´ 39´´
20	CALY	Mexico, Oaxaca, San Lorenzo Albarradas	OAX	XTP	11	1840	16° 52´ 58´´	96° 16´ 06´´
21	CALY	Mexico, Oaxaca, Sta. María Albarradas	OAX	XTF	13	1780	16° 58´ 09´´	96° 11´ 31´´
22	CALY	Mexico, Oaxaca, Col. Emiliano Zapata	OAX	XTF	2	1484	16° 45´ 07´´	96° 50´ 42´´
23	CALY	Mexico, Oaxaca, San Baltazar Guelavía	OAX	XTF	1	1718	16° 47´ 34´´	96° 20´ 02´´
24	CALY	Mexico, Oaxaca, Sola de Vega	OAX	XTF	9	1430	16° 30´ 39´´	96° 58´ 57´´
25	CALY	Mexico, Oaxaca, Macahuite	OAX	TDF	2	54	15° 43´ 32´´	96° 39´ 48´´

26	CALY	Mexico, Oaxaca, Santiago Matatlán	OAX	XTF	11	1784	16° 50´ 53´´	96° 22´ 18´´
27	CALY	Mexico, Oaxaca, Tuxtepec, Ixtlán	OAX	XTF	5	1993	17° 19´ 28´´	96° 29´ 09´´
28	CALY	Mexico, Oaxaca, Tuxtepec, Puente Xia	OAX	XTF	5	1513	17° 18´ 19´´	96° 31´ 35´´
29	SCHI	Mexico, Oaxaca, Santiago Comaltepec	OAX	CF	10	848	17° 41´ 23´´	96° 20´ 13´´
30	SCHI	Mexico, Veracruz, Actópan	SMOr	TDF	10	322	19º 23´ 13´´	96º 36´ 56´´
31	SCHI	Mexico, Veracruz, Cardel, La Mancha	SMOr	TDF	6	61	19° 36´ 02´´	96° 22´ 29´´
32	SCHI	Mexico, Veracruz, Xico	SMOr	CF	12	1350	19º 24´ 37´´	96º 59´ 31´´
33	SCHI	Mexico, Veracruz, El Riscal	SMOr	CF	11	1586	19º 28´ 47´´	96º 59´ 51´´
34	SCHI	Mexico, Veracruz, La Pitaya	SMOr	CF	13	1343	19º 30´ 27´´	96º 57´ 39´´
35	SCHI	Mexico, Veracruz, Clavijero	SMOr	CF	14	1225	19º 30´ 47´´	96º 56´ 28´´
36	SCHI	Mexico, Veracruz, Rancho Viejo	SMOr	CF	13	1350	19º 31´ 11´´	96º 58´ 22´´
37	SCHI	Mexico, Veracruz, Coapexpan	SMOr	CF	10	1392	19º 31´ 22´´	96º 58´ 02´´
38	SCHI	Mexico, Veracruz, Xoloxtla	SMOr	CF	10	1454	19º 31´ 36´´	97º 00´ 34´´
39	SCHI	Mexico, Veracruz, Las Minas	SMOr	CF	9	1828	19º 34´ 17´´	96º 59´ 21´´
40	SCHI	Mexico, Veracruz, Tlalnelhuayocan	SMOr	CF	6	1624	19º 34´ 47´´	96º 57´ 38´´
41	SCHI	Mexico, Veracruz, Coacoatzintla	SMOr	CF	11	1501	19º 37´ 41´´	96º 52´ 56´´
42	SCHI	Mexico, Veracruz, Volcán de Acatlán	SMOr	CF	8	1840	19º 40´ 47´´	96º 51´ 11´´
43	SCHI	Mexico, Puebla, Lagunillas	SMOr	CF	6	1500	20° 13´ 23´´	97° 57´ 33´´
44	SCHI	Mexico, San Luis Potosí, Xilitla	SMOr	TDF	9	836	21° 21´ 39´´	98° 59´ 35´´
45	SCHI	Mexico, Veracruz, Las Choapas	SMOr	TDF	1	18	17° 38´ 41´´	93° 58´ 54´´
46	SCHI	Mexico, Chiapas, Jitotol	CHIS	CF	4	1698	17° 02´ 54´´	92° 51´ 18´´
47	SCHI	Mexico, Chiapas, Ocosingo	CHIS	TDF	6	876	16° 55´ 06´´	92° 06´ 09´´
48	CALY	Mexico, Chiapas, Arriaga, La Aurora	CHIS	TDF	4	699	16° 28´ 18´´	93° 49´ 55´´
49	CALY	Mexico, Chiapas, Motozintla	CHIS	TDF	6	1353	15° 21´ 21´´	92° 14´ 54´´
50	CALY	Mexico, Chiapas, Ciudad Cuauhtemoc	CHIS	TDF	9	1834	15° 55´ 04´´	91° 58´ 55´´
51	CALY	Mexico, Chiapas, Parque Ya' Ax-Na	CHIS	TDF	6	1595	16° 13´ 24´´	92° 07´ 48´´

Region and habitat abbreviations are as follows: CHIS = Chiapas; OAX = Oaxaca; SMOr = Sierra Madre Oriental; TMVB = Trans-Mexican Volcanic Belt; CF = cloud forests; XTF = xeric temperate forests; TDF = tropical dry forests.

Table S2. Genetic variability and summary statistics (mean ± SD) of *Psittacanthus calyculatus* / *schiedeanus* complex populations grouped based on previous species assignments (A), habitat type (B), admixture level (C) or mountain geography (D). Data estimated for nine loci.

Classification	Sample size	Total number of alleles	Allelic richness	Mean rarefied allelic richness	Observed Heterozygosity (H_o)	Expected Heterozygosity (H_e)	Inbreeding Coefficient (F_{is})
(A) Species							
CALY	215	177	17.38	17.15	0.29	0.81	0.63 (0.61–0.66)
SCHI	192	145	14.6	13.74	0.43	0.76	0.43 (0.39–0.46)
(B) Habitat							
CF	126	127	11.15	13.29	0.45	0.73	0.38 (0.34–0.42)
XTF	223	169	13.21	12.26	0.28	0.8	0.64 (0.61–0.66)
TDF	58	118	11.47	11.11	0.37	0.83	0.55 (0.48–0.62)
(C) Admixture groups							
CALY	135	122	11.61	6.2	0.26	0.7	0.62 (0.57-0.66)
HYBR	114	152	15.18	8.09	0.32	0.83	0.61 (0.56-0.66)
SCHI	158	140	13.48	6.72	0.44	0.75	0.41 (0.37-0.45)
(D) Geography							
CHIS	35	90	8.49	6.33	0.33	0.82	0.59 (0.49–0.69)
OAX	90	130	10.26	6.35	0.30	0.78	0.61 (0.57–0.66)
SMOr	147	132	9.89	5.64	0.45	0.74	0.39 (0.35–0.43)
TMVB	135	122	8.68	5.39	0.26	0.70	0.62 (0.57–0.66)

F_{IS}= Parenthesis include the 95% CI estimated with 1000 bootstraps in the R package DiveRsity[91].

Table S3. Results of the AMOVA models on *Psittacanthus schiedeanus*/*calyculatus* populations grouped by (A) species (*calyculatus*, *schiedeanus*), (B) habitat type (CF, XTF, TDF), (C) admixture level (CALY, HYBR, SCHI), and (D) mountain geography (CHI, OAX, SMOr, TMVB).

	d.f.	Sum of squares	Variance components	Percentage of variation	Fixation indices
(A) Species					
Among groups	1	76.26	0.1614	5.83	F_{ST} = 0.2605***
Among populations within groups	49	535.91	0.5599	20.23	F_{SC} = 0.2148***
Within populations	765	1565.91	2.0400	73.94	F_{CT} = 0.0583***
(B) Habitat type					
Among groups	2	102.97	0.1641	5.97	F_{ST} = 0.25582***
Among populations within groups	48	509.21	0.5395	19.61	F_{SC} = 0.20859***
Within populations	765	1565.91	2.0495	74.42	F_{CT} = 0.05968***
(C) Admixture					
Among groups	2	115.07	0.1691	6.67	F_{ST} = 0.2532***
Among populations within groups	48	497.11	0.5248	19.75	F_{SC} = 0.20407***
Within populations	765	1565.91	2.0469	74.68	F_{CT} = 0.06173***
(D) Geography					
Among groups	3	132.65	0.1746	6.38	F_{ST} = 0.25170***
Among populations within groups	47	479.53	0.5139	18.79	F_{SC} = 0.20069***
Within populations	765	1565.91	2.0469	74.83	F_{CT} = 0.06382***

Table S4. Posterior parameter estimates for the best-supported scenario (scenario 2, *isolation with admixture model* in which HYBR population was generated by admixture between CALY and SCHI populations at t1, then CALY merged with SCHI at t2) considering the three mistletoe groups (CALY, CHIS, HYBR). Estimates are based on 1% of simulated datasets closest to the observed values. Simulations and approximate Bayesian computation (ABC) analyses were performed considering microsatellites.

Parameter	Mean	Median	Mode	q025	q050	q250	q750	q950	q975
CALY	9.15E+03	8.25E+03	7.46E+03	2.88E+03	3.49E+03	5.98E+03	1.12E+04	1.83E+04	2.16E+04
HYBR	2.08E+04	2.13E+04	2.36E+04	9.44E+03	1.10E+04	1.67E+04	2.54E+04	2.90E+04	2.95E+04
SCHI	1.37E+04	1.34E+04	1.31E+04	6.19E+03	7.27E+03	1.08E+04	1.63E+04	2.09E+04	2.30E+04
t1	9.01E+03	8.28E+03	6.66E+03	2.77E+03	3.34E+03	5.83E+03	1.15E+04	1.73E+04	1.91E+04
t2	1.75E+04	1.80E+04	2.30E+04	7.68E+03	8.94E+03	1.40E+04	2.15E+04	2.43E+04	2.47E+04
ra	5.47E-01	5.64E-01	6.23E-01	5.44E-02	1.04E-01	3.69E-01	7.37E-01	9.31E-01	9.64E-01
Mean mutation rate (Aμmic_1)	5.04E-04	4.77E-04	3.94E-04	1.81E-04	2.13E-04	3.50E-04	6.42E-04	8.78E-04	9.33E-04
Mean coeff. (pmic_1)	2.37E-01	2.48E-01	3.00E-01	1.23E-01	1.36E-01	2.03E-01	2.80E-01	3.00E-01	3.00E-01
Mean SNI rate (snimic_1)	1.48E-06	5.92E-07	1.29E-08	1.53E-08	2.10E-08	1.27E-07	2.02E-06	6.04E-06	7.45E-06

Parameters are *N* = effective population size for CALY (pop1, N1), HYBR (Pop2, N2), SCHI (Pop3, N3), *t* = time since divergence, ra = admixture rate and μ = mutation rate for nuclear microsatellites. Group abbreviations are as follows: CALY = *P. calyculatus* along the TMVB; HYBR = admixed region (Oaxaca and Chiapas); SCHI = *P. schiedeanus* along the Sierra Madre Oriental.

Fig. S1 Admixture proportions obtained with STRUCTURE for (A) simulated hybrid individuals and (B) individuals from natural populations of the *Psittacanthus calyculatus* / *P. schiedeanus* complex.

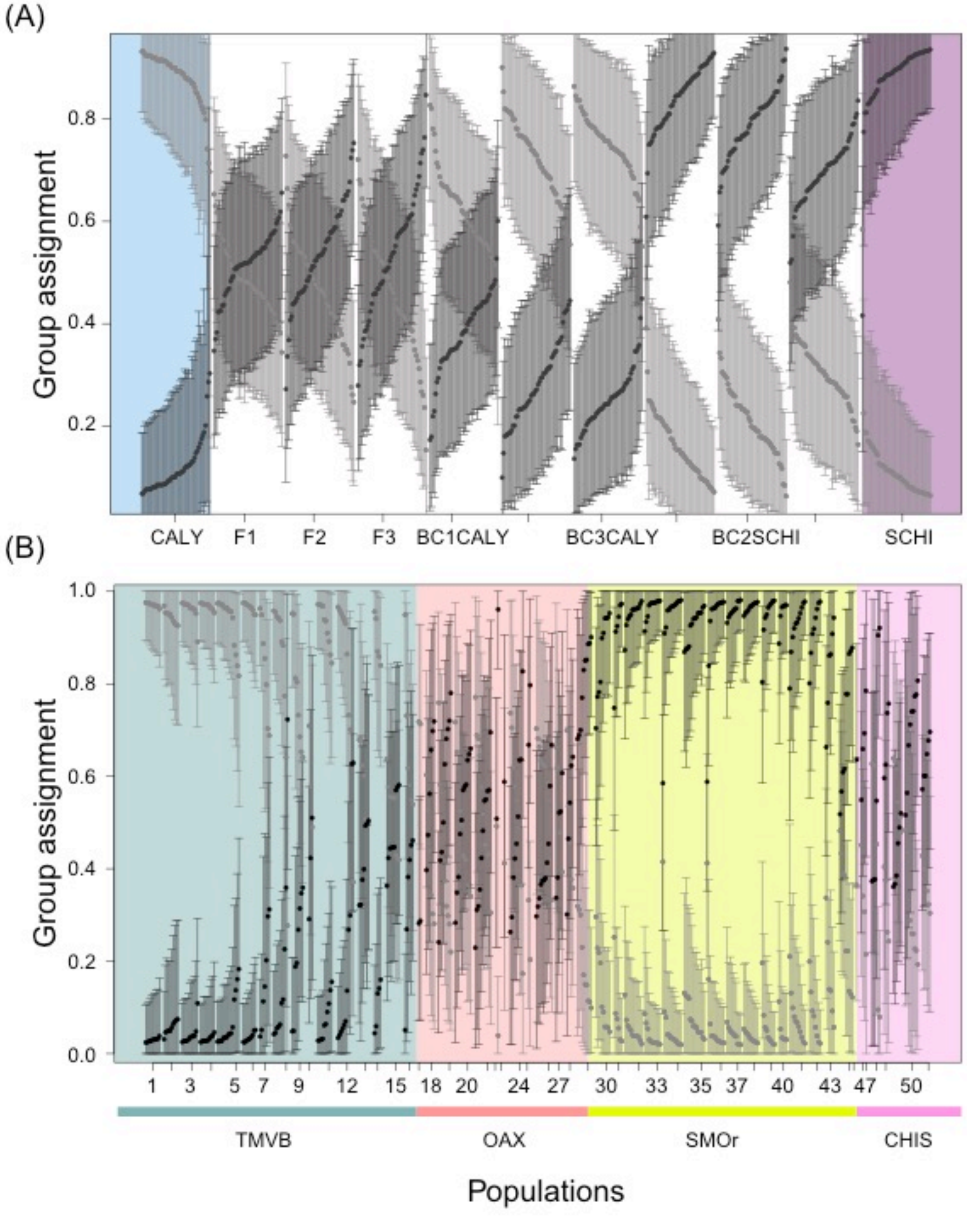

Supplement: Supplementary file 1 — Supplementary Information [file 41598_2018_23707_MOESM1_ESM.pdf]
